# Transanal minimally invasive proctectomy for ulcerative colitis is beneficial in terms of short‐term outcomes and defecation function

**DOI:** 10.1002/ags3.12844

**Published:** 2024-07-14

**Authors:** Marie Hanaoka, Yusuke Kinugasa, Kenta Yao, Ayumi Takaoka, Megumi Sasaki, Shinichi Yamauchi, Masanori Tokunaga

**Affiliations:** ^1^ Department of Gastrointestinal Surgery Tokyo Medical and Dental University Bunkyo‐ku Tokyo Japan

**Keywords:** defecation function, ileal pouch‐anal anastomosis, transanal minimally invasive proctectomy, ulcerative colitis

## Abstract

**Objective:**

Despite being reported safety, the advantages of transanal minimally invasive proctocolectomy (TAMIP) are controversial, and comparative studies on postoperative defecation function between ileal pouch‐anal anastomosis (IPAA) using laparoscopic transanal manipulation (TAMIP‐IPAA) and without this technique (traditional IPAA) are lacking. This study analyzed TAMIP's impact on short‐term and postoperative defecation function in patients with ulcerative colitis (UC) to evaluate its safety and feasibility.

**Methods:**

Inclusion criteria comprised patients with UC undergoing minimally invasive proctocolectomy at our hospital from May 2014 to May 2023. The TAMIP‐IPAA approach involved precise rectal mucosa removal while preserving the sphincter muscle during laparoscopic transanal manipulation.

**Results:**

In the evaluation of short‐term outcomes for 71 patients undergoing proctocolectomy, the TAMIP group (37 patients) outperformed the non‐TAMIP group in operative time (395 vs. 289 min, *p* < 0.001) and postoperative hospital stay (12 vs. 8 days, *p* < 0.001). Additionally, TAMIP‐IPAA demonstrated advantages over traditional IPAA (seven patients), in operative time (443 vs. 289 min, *p* = 0.006), intraoperative blood loss (392 vs. 130 mL, *p* = 0.001), postoperative hospital stay (18 vs. 8 days, *p* = 0.003), anastomotic leakage (42.9% vs. 8.1%, *p* = 0.041), and re‐admission within 30 days (57.1% vs. 8.1%, *p* = 0.009). Wexner scores were significantly superior in the TAMIP‐IPAA group at 6 months (14.5 vs. 8.0 points, *p* = 0.029) and 1 year post stoma closure (14.0 vs. 7.0 points, *p* = 0.020), indicating enhanced short‐term outcomes and defecation function compared to traditional IPAA.

**Conclusions:**

TAMIP‐IPAA for UC has the potential to offer promising benefits, including the enhancement of short‐term outcomes and the improvement of defecation function.

## Graphical abstract

The transanal minimally invasive proctocolectomy approach during proctocolectomy for UC has the potential to offer promising benefits, including the enhancement of short‐term outcomes and the improvement of defecation function.

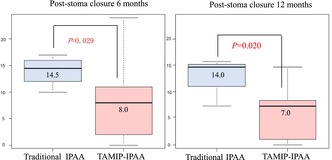

## 1 INTRODUCTION

Handsewn ileal pouch‐anal anastomosis (IPAA) or stapled IPAA (ileal pouch‐anal canal anastomosis [IACA]) is the established surgical procedure for managing ulcerative colitis (UC) in patients with fulminant/severe or refractory disease, or inflammation‐induced cancer. Complete anal mucosal resection with handsewn IPAA eliminates the risk of carcinogenesis from residual rectal mucosa, making it widely accepted as the most effective treatment with a low risk of residual rectal cuffs longer than 2 cm, thereby preventing long‐term pouch dysfunction.^1^ Given that many patients with UC are young and have specific life goals, including raising children and participating in society, a swift return to their normal quality of life after surgery is crucial. This necessitates avoiding the risk of long‐term pouch dysfunction and enhancing short‐term outcomes.^1^, ^2^, ^3^, ^4^

In conventional laparoscopic surgery, operability is hindered by interference from forceps deep within the pelvis. To overcome this limitation, new minimally invasive surgeries, including transanal laparoscopic approaches, have been developed; these approaches, previously utilized for transanal minimally invasive surgery (TAMIS) and transanal total mesorectal excision (TaTME) in rectal cancer,^5^ were first reported for deep pelvic manipulation during proctocolectomy for UC in 2015.^6^ Recent reports have highlighted the short‐term safety and feasibility of the transanal minimally invasive approach for IPAA with proctocolectomy in patients with UC.^4^, ^7^, ^8^, ^9^ However, the merits of this approach are still controversial, and to date, there have been no reports comparing postoperative defecation function between minimally invasive transabdominal and transanal approaches to IPAA.

Our emphasis is on the transanal laparoscopic approach for UC, prioritizing mucosal removal and preservation of the anal sphincter. Since 2017, we have actively undertaken IPAA through a method known as transanal minimally invasive proctectomy (TAMIP), where the transanal operation is performed laparoscopically.

In this study, we aimed to assess the impact of TAMIP on postoperative outcomes and defecation function following proctocolectomy for UC.

## 2 MATERIALS AND METHODS

### 2.1 Target cases, approaches, and reconstruction methodology

#### 2.1.1 Target cases

This retrospective cohort study included consecutive patients who underwent minimally invasive proctocolectomy with a one‐stage or two‐stage approach for UC at our hospital from May 2014 to May 2023. Fulminant and severe cases treated with the three‐stage approach (subtotal colectomy in the first stage, residual rectal resection in the second stage, and stoma closure in the third stage) were excluded due to significantly different short‐term outcomes. The TAMIP approach, enabling two‐team surgery and reducing operative time, was used for handsewn IPAA and abdominoperineal resection (APR) cases. Our standard surgical protocol for UC involved two‐stage surgery (stapled IPAA or handsewn IPAA in the first stage, and stoma closure in the second stage). Cases where anastomosis was highly risky or undesired underwent proctocolectomy with APR from the beginning.

The analysis compared postoperative outcomes between non‐TAMIP and TAMIP groups. Additionally, defecation function was compared between traditional IPAA (laparoscopic handsewn IPAA) and TAMIP‐IPAA. The study was approved by the ethics committee of our institution (approval number M2020‐367) and followed the Strengthening the Reporting of Observational Studies in Epidemiology (STROBE) guidelines. Informed consent was obtained from the patients. An opt‐out form on the study website, which has been approved by the Ethics Committees, was used when informed consent could not be obtained.

#### 2.1.2 Approaches

In patients scheduled for handsewn IPAA, an initial mini‐laparotomy assessed the feasibility of handsewn IPAA using the planned J‐pouch. If handsewn IPAA was unfeasible, stapled IPAA was performed. If feasible in TAMIP cases, transabdominal and transanal manipulation were simultaneously initiated by two teams.

The TAMIP approach was utilized for patients scheduled for both IPAA and APR procedures. For both cases, transanal manipulation, GelPoint® Path (Applied Medical, Rancho Santa Margarita, CA, USA), and AirSeal (CONMED, Tokyo, Japan) were used to maintain continuous pneumoperitoneum and enhance visibility.

In TAMIP approach, following mucosal resection from the dentate line, the internal anal sphincter and the longitudinal muscle layer was carefully dissected after preserving a 1–2 cm rectal cuff^9^ in cases with mild proctitis or cases with no dysplasia.^10^ After dissecting the anorectal ligament at the 6 o'clock position, the dissection plane was expanded dorsally. Then, the loose dissection plane at the 2 and 10 o'clock positions was further expanded at the 12 o'clock position. The communication point between the abdominal and transanal dissection planes was facilitated by each team.

In non‐TAMIP, traditional IPAA (employing conventional laparoscopic abdominal techniques with direct transanal visualization) and stapled IPAA (utilizing conventional laparoscopic or robotic‐assisted approach) were included and performed by a single team.

While our preference leaned towards TAMIP for its short‐term outcome benefits, stapled IPAA was chosen in cases where the ileal pouch would not reach the anus due to one of the following factors: abundant visceral fat, tall stature, a narrow pelvis, or diffuse adhesions from previous surgery.^11^, ^12^ To address this, considering past reports, high body mass index (BMI)^13^, ^14^ was selected as the determinant for the choice of anastomosis. A BMI cutoff of 25 kg/m^2^ was established based on past experiences, assigning cases with BMI ≥25 kg/m^2^ to stapled IPAA and those with BMI <25 kg/m^2^ to handsewn IPAA. This criterion eliminated the occurrence of cases where the ileal pouch could not reach the anus.

The selection between robotic‐assisted and conventional laparoscopic approach in stapled IPAA was made during a preoperative conference within the department. Robotic‐assisted stapled IPAA allows precise pelvic manipulation and deep rectal mobilization up to the anal canal, so that it was selected for cases with severe colitis extending to the anal canal, or for residual rectal resection in the second stage of a three‐stage surgery, which predominantly involves intrapelvic manipulation.

#### 2.1.3 Reconstruction methodology

For handsewn IPAA or stapled IPAA, the specimen was removed from a site of mini‐laparotomy, and an ileal pouch (J‐pouch) was created. Subsequently, the tip of the J‐pouch was guided to the pelvic floor.

In cases of stapled IPAA, the anastomosis was created using the double stapling technique. Handsewn IPAA includes traditional IPAA and TAMIP‐IPAA. In traditional IPAA, the rectum was mobilized as deeply as possible under laparoscopic abdominal vision, and its mucous membrane was then removed transanally under direct vision, connecting it to the abdominal cavity. In TAMIP‐IPAA cases, the technique of rectal mucosa removal and sphincter preservation is described above.

In all cases with a temporary ileostomy, loop ileostomies were used. The timeframe between the first to the second stage (ileostomy closure) of the two‐stage surgery was, as a rule, set to 3 months, not exceeding 12 months.

#### 2.1.4 Evaluation methods

**FIGURE 1:**
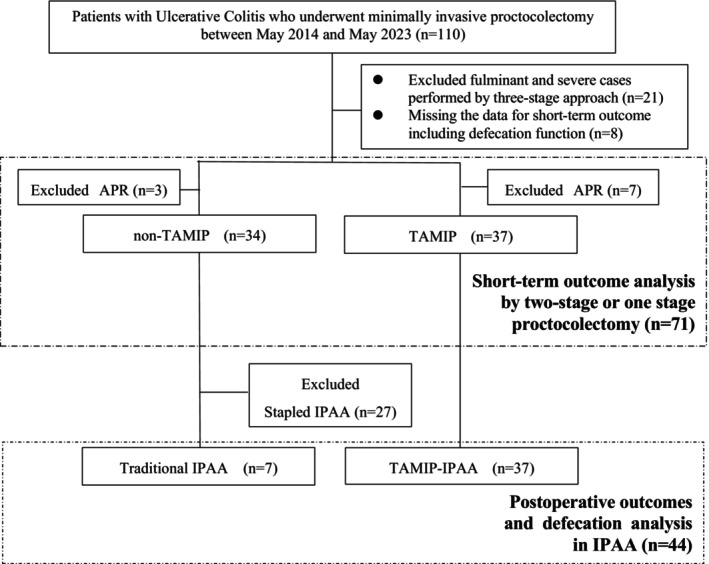
Flow diagram of the study. APR, abdominoperineal resection; IPAA, ileal pouch‐anal anastomosis; TAMIP, transanal minimally invasive proctectomy.

Initially, we compared the short‐term outcomes of non‐TAMIP vs. TAMIP in proctocolectomy (Figure 1). Subsequently, we compared postoperative outcomes and defecation function between traditional IPAA and TAMIP‐IPAA (Figure 1).

Regarding postoperative outcomes, we extracted data from medical records on age, sex, BMI, American Society of Anesthesiologists Physical Status (ASA‐PS), UC duration, UC treatment history, type of surgery, indication for surgery, preoperative serum albumin level, reconstruction method, operative time, amount of blood loss, and postoperative complications. Postoperative complications (within 60 days after each surgery), such as anastomotic leakage, bleeding, small bowel obstruction, surgical site infection after stoma closure, and others, were categorized and assessed using the Clavien–Dindo (CD) classification.^15^ We conducted a questionnaire survey on defecation function in daily clinical practice for all patients who underwent proctocolectomy followed by stoma closure and reported in the medical record. After the initiation of this study, questionnaires were distributed and collected using the system described below. Patients voluntarily submitted completed questionnaires to a designated secretary for outpatients (an external third party in this study) preoperatively and at regular outpatient clinic visits at 6, 12, and 24 months after ileostomy closure, ensuring the absence of surgeon observation. The Cleveland Clinic Florida‐Fecal Incontinence Score (Wexner score) and defecation frequency were recorded.

The definition of pouch failure was established as either late stoma reversal or non‐reversal of the stoma, and this observation period was set up to 10 years after the final surgery.

#### 2.1.5 Statistical analysis

Categorical variables are presented as numbers (%), while continuous variables are expressed as median and range. To assess differences between groups, Fisher's exact test was used for categorical variables, and the Mann–Whitney U test was used for continuous variables. Statistical analysis was performed by MH, who possesses statistical expertise, with the support of a statistician.

## 3 RESULTS

### 3.1 Patient flow

Figure 1 shows the patient flow of this study. Initially, of the 110 patients who underwent proctocolectomy for UC at our hospital from May 2014 to May 2023, 21 fulminant/severe cases treated with the three‐stage approach (*n* = 21), eight lacking short‐term outcomes, including defecation function, and 10 patients who underwent APR were excluded. Consequently, 71 patients were included in the assessment of short‐term outcomes after proctocolectomy: 34 non‐TAMIP and 37 TAMIP. After excluding those with reconstruction methods other than handsewn IPAA from the non‐TAMIP group (27 stapled IPAA cases), the 44 handsewn IPAA patients were divided into traditional IPAA (*n* = 7) and TAMIP‐IPAA (*n* = 37) groups for postoperative outcome and defecation function evaluation.

### 3.2 Patient characteristics of TAMIP versus non‐TAMIP

Table 1 outlines the characteristics of the 71 patients assessed for short‐term outcomes of TAMIP vs. non‐TAMIP. Median age was 55 years old in the non‐TAMIP group and 35 in the TAMIP group (*p* = 0.002). No significant differences existed in BMI, ASA‐PS, and preoperative serum albumin levels, duration of UC, history of UC treatment, and surgery indication. Emergency surgery was more common in the TAMIP group (48.6%) than in the non‐TAMIP group (14.7%; *p* = 0.003). In the TAMIP group, all cases were reconstructed using handsewn anastomosis, whereas in the non‐TAMIP group, the stapled technique was predominant (79.4%) (*p* < 0.001).

**TABLE 1:** Comparison of patients' background characteristics between non‐TAMIP and TAMIP approaches in proctocolectomy.

Characteristics	Non‐TAMIP	TAMIP	p
	*n* = 34	*n* = 37	
Age (years)*	55 (17–73)	35 (15–58)	0.002
Sex (male)	25 (73.5)	23 (62.2)	0.616
BMI (kg/m^2^)*	20 (14–33)	19 (14–27)	0.068
ASA‐PS (I/II/III)	2/29/3	1/33/3	1.000
Serum albumin (g/dL)*	3.2 (1.5–4.6)	3.2 (1.8–4.5)	0.915
Duration of UC (years)*	12 (0–34)	6 (0–33)	0.884
Past treatment			
Use of steroid	25 (73.5)	27 (73.0)	1.000
Use of TNF antibody	19 (55.9)	25 (67.6)	0.470
Use of steroid and TNF antibody	19 (55.9)	24 (64.9)	0.475
Use of LCAP	5 (14.7)	7 (18.9)	0.756
Indication for surgery			
UCAN or dysplasia	16 (47.1)	12 (32.4)	0.233
Resistance to treatment	18 (52.9)	25 (67.6)	
Emergency operation	5 (14.7)	18 (48.6)	0.003
Approach			
Laparoscopic	29 (85.3)	36 (97.3)	0.098
Robotic	5 (14.7)	1 (2.7)	
Re‐construction			
Stapled IPAA	27 (79.4)	0	<0.001
Handsewn IPAA	7 (20.6)	37 (100)	
Anastomosis AV (cm)*	3.0 (0.5–8.0)	0.5 (0.5–2.0)	<0.001

Abbreviations: ASA‐PS, American Society of Anesthesiologists Physical Status; AV, anal verge; BMI, body mass index; IPAA, ileal pouch‐anal anastomosis; LCAP, leukocytapheresis; TAMIP, transanal minimally invasive proctectomy; TNF, tumor necrosis factor; UC, ulcerative colitis; UCAN, ulcerative colitis associated neoplasia.

* Median, range.

### 3.3 Short‐term outcomes of non‐TAMIP versus TAMIP

Table 2 illustrates the short‐term outcomes of proctocolectomy. The TAMIP group showed significantly shorter operative time (395 vs. 289 min, *p* < 0.001) and postoperative hospital stay (12 vs. 8 days, *p* < 0.001) compared to the non‐TAMIP group. No significant differences were observed in blood loss (122 vs. 130 mL, *p* = 0.804) and the incidence of morbidity of CD grade ≥II (32.4% vs 29.7%, *p* = 0.207), respectively.

**TABLE 2:** Comparison of short‐term outcomes between non‐TAMIP and TAMIP approaches in proctocolectomy.

Characteristics	Non‐TAMIP	TAMIP	*p*
	*n* = 34	*n* = 37	
Operative time (min) ^a^	395 (244–715)	289 (189–658)	<0.001
Blood loss (mL) ^a^	122 (0–1164)	130 (0–905)	0.804
Morbidity^a^ CD grade ≥ II	11 (32.4)	11 (29.7)	0.207
CD II/III	9 (26.5)/2 (5.9)	8 (21.6)/3 (8.1)	1.000
Anastomotic leakage	4 (11.8)	3 (8.1)	0.703
Small bowel obstruction	5 (14.7)	4 (10.8)	1.000
Pouchitis	3 (8.8)	8 (21.6)	0.193
Dehydration	0	1 (2.7)	1.000
Urinary retention	0	0	1.000
Wound infection	0	1 (2.7)	1.000
Others	1 (2.9)	2 (5.4)	1.000
Postoperative hospital stay^b^	12 (6–42)	8 (6–25)	<0.001
Re‐admission within 30 days	5 (14.7)	3 (8.1)	0.703
Pouch failure	3 (8.8)	3 (8.2)	1.000
Mortality	0	0	1.000

Abbreviations: CD, Clavien–Dindo classification; TAMIP, Transanal minimally invasive proctectomy.

^a^ Within 30 days after surgery.

^b^ Median, range.

### 3.4 Patient characteristics and short‐term outcomes of traditional IPAA versus TAMIP‐IPAA

We then analyzed the 44 cases undergoing handsewn IPAA; the traditional IPAA and the TAMIP‐IPAA groups consisted of seven and 37 patients, respectively (Table 3). TAMIP‐IPAA was the same cohort as the TAMIP group because the re‐constriction method of the TAMIP group was handsewn in all cases. The groups exhibited no significant differences in age, sex, BMI, ASA‐PS, preoperative serum albumin level, duration of UC, rate of emergency surgery, and surgical indication.

**TABLE 3:** Comparison of patients' background characteristics and short‐term outcomes between traditional IPAA and TAMIP‐IPAA approaches.

Characteristics	Traditional IPAA	TAMIP‐IPAA	*p*
	*n* = 7	*n* = 37	
Age (years)*	52 (24–66)	35 (15–58)	0.064
Sex (male)	3 (42.8)	23 (62.2)	0.419
BMI (kg/m^2^)*	19 (14–29)	19 (14–27)	0.576
ASA‐PS (I/II/III)	0/6/1	1/33/3	0.542
Serum albumin (g/dL)*	3.5 (2.6–4.2)	3.2 (1.8–4.5)	0.103
Duration of UC (years)*	18 (2–29)	6 (0–33)	0.037
Emergency operation	2 (28.6)	18 (48.6)	0.420
Approach			
Laparoscopic	7 (100)	36 (97.3)	1.000
Robotic	0	1 (2.7)	
Indication for surgery			
UCAN or dysplasia	3 (42.9)	12 (32.4)	0.404
Resistance to treatment	4 (57.1)	25 (67.6)	0.675
Operative time (min)*	443 (331–626)	289 (189–658)	0.006
Blood loss (mL)*	392 (85–686)	130 (0–905)	0.001
Morbidity^b^ CD grade ≧II	4 (57.1)	11 (29.7)	0.207
CD II/III	4/0	8/3	0.516
Anastomotic leakage	3 (42.9)	3 (8.1)	0.041
Small bowel obstruction	2 (28.6)	4 (10.8)	0.248
Postoperative hospital stay*	18 (10–42)	8 (6–25)	0.003
Re‐admission within 30 days	4 (57.1)	3 (8.1)	0.009
Pouch failure	2 (28.6)	3 (8.2)	0.173
Mortality	0	0	1.000

*Note*: Data are given as number of cases (%). Continuous variables are given as median (range).

Abbreviations: ASA‐PS, American Society of Anesthesiologists Physical Status; BMI, body mass index; CD, Clavien–Dindo classification; IPAA, ileal pouch‐anal anastomosis; TAMIP, transanal minimally invasive proctectomy; UC, ulcerative colitis; UCAN, ulcerative colitis associated neoplasia.

* Median, range.

Regarding short‐term outcomes, the TAMIP‐IPAA group exhibited significantly shorter operative time (traditional IPAA vs. TAMIP‐IPAA = 443 vs. 289 min, *p* = 0.006), decreased blood loss (392 vs. 130 mL, *p* = 0.001), and shorter postoperative hospital stay (18 vs. 8 days, *p* = 0.003). The TAMIP‐IPAA group showed significantly lower rates of anastomotic leakage (42.9% vs. 8.1%, *p* = 0.041) and re‐admission within 30 days (57.1% vs. 8.1%, *p* = 0.009).

Regarding pouch failure, the median follow‐up periods were 6 years for the traditional IPAA and 4 years for the TAMIP‐IPAA group. The pouch failure rate was 28.6% (2/7) in the traditional IPAA group and 8.2% (3/37) in the TAMIP‐IPAA group (*p* = 0.173), indicating no significant difference; nonetheless, the TAMIP‐IPAA group exhibited a lower rate. In the traditional IPAA group, pouch failure cases involved intractable fistulas due to delayed anastomotic leakage and anastomotic stenosis, occurring over 1‐year post‐stoma closure, necessitating planned surgery after unsuccessful medical management. In the TAMIP group, two cases developed pelvic sepsis due to severe pouchitis as late complications, leading to ileostomy construction, as a fulminant disease condition. Additionally, one case in the TAMIP involved lung metastasis, not undergoing stoma closure postoperatively.

### 3.5 Postoperative defecation function between traditional IPAA versus TAMIP‐IPAA

**FIGURE 2:**
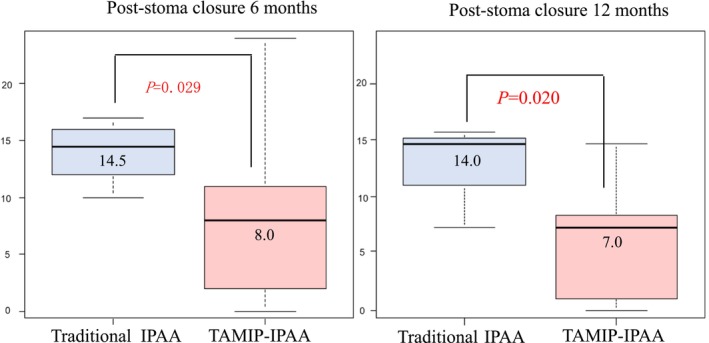
Comparison of Wexner Scores at 6 and 12 months after traditional IPAA vs. TAMIP‐IPAA. At 6 months post‐stoma closure, the Wexner scores for the TAMIP‐IPAA and traditional IPAA groups were 8.0 and 14.5, respectively (*p* = 0.029). At 1‐year post‐stoma closure, the scores were 7.0 and 14.0, respectively (*p* = 0.020). Boxes indicate interquartile ranges. Bold lines are the medians, and bars are the ranges of scores. IPAA, ileal pouch‐anal anastomosis; TAMIP; transanal minimally invasive proctectomy.

**FIGURE 3:**
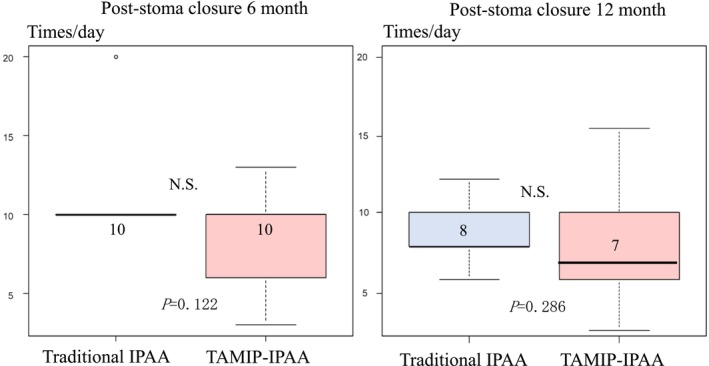
Comparison of defecation frequency at 6 and 12 months after traditional IPAA vs. TAMIP‐IPAA. At 6 months post‐stoma closure, defecation frequencies were 10 times/day in both groups. At 1‐year post‐stoma closure, the frequencies were eight times/day for the traditional IPAA group and seven times/day for the TAMIP‐IPAA group (*p* = 0.286). Boxes indicate interquartile ranges. Bold lines are the medians, and bars are the ranges of scores. IPAA, ileal pouch‐anal anastomosis; N.S., not significant; TAMIP, transanal minimally invasive proctectomy.

Postoperative defecation function details are outlined in Table 4 and depicted in Figures 2 and 3. At 6 months post‐stoma closure, the TAMIP‐IPAA group showed significantly better results, with a Wexner score of 8.0 compared to 14.5 in the traditional IPAA group (*p* = 0.029). At 1‐year post‐stoma closure, the TAMIP‐IPAA group continued to demonstrate superior outcomes, with a Wexner score of 7.0 compared to 14.0 in the traditional IPAA group (*p* = 0.020) (Figure 2). Regarding defecation frequency, both the traditional IPAA and TAMIP‐IPAA groups reported 10 times/day at 6 months post‐stoma closure, with no significant difference. At 1‐year post‐stoma closure, defecation frequencies were eight times/day for traditional IPAA and seven times/day for TAMIP‐IPAA, showing a slight advantage for the TAMIP‐IPAA group (*p* = 0.286).

**TABLE 4:** Comparison of defecation function between traditional IPAA and TAMIP‐IPAA.

Characteristics	Traditional IPAA	TAMIP‐IPAA	*p*
	*n* = 7	*n* = 37	
Wexner score at 6 months after stoma closure	14.5 (10.0–17.0)	8.0 (0–11.0)	0.029
Wexner score at 12 months after stoma closure	14.0 (7.0–15.0)	7.0 (0–14.0)	0.020
Frequency of bowel movements at 6 months after stoma closure (times/day)	10 (10–20)	10 (3–13)	0.122
Frequency of bowel movements at 12 months after stoma closure (times/day)	8 (6–12)	7 (3–15)	0.286

*Note*: All data are presented as median and range.

Abbreviations: IPAA, ileal pouch‐anal anastomosis; TAMIP, transanal minimally invasive proctectomy.

## 4 DISCUSSION

This study systematically compared short‐term outcomes between TAMIP and non‐TAMIP approaches in proctocolectomy for UC, as well as postoperative outcomes and defecation function in TAMIP‐IPAA vs. traditional IPAA within handsewn IPAA. First, TAMIP demonstrated superiority over non‐TAMIP in proctocolectomy, with advantages in operative time and postoperative hospital stay. Additionally, TAMIP‐IPAA outperformed traditional IPAA across multiple parameters, including operative time, blood loss, postoperative hospital stay, anastomotic leakage, and re‐admission within 30 days. Consistently, the TAMIP‐IPAA group exhibited significantly better Wexner scores at both 6 months and 1 year post stoma closure, indicating improvements not only in short‐term outcomes but also in defecation function compared to traditional IPAA.

Although widely accepted for rectal cancer,^16^, ^17^, ^18^ there is limited evidence comparing the minimally invasive transanal approach, specifically TAMIP, to conventional laparoscopic methods in proctocolectomy for UC.^4^, ^6^ Previous literature primarily consists of case reports or case series, emphasizing the scarcity of rigorous comparisons.^9^ The present study fills this gap, showing that TAMIP significantly reduces operative time. In particular, the median operative time was reduced by more than 100 min, a clinically significant improvement that minimizes the invasiveness to the patient.

Additionally, a few studies^19^, ^20^ have reported long‐term complications after TAMIP‐IPAA, and there is no comparison with traditional IPAA. In this study, although there was no significant difference, the rate of pouch failure was lower with TAMIP‐IPAA than with traditional IPAA (8.2% vs 28.6%), which is an important outcome for the patient's long‐term quality of life.

Remarkably, our study is the first to compare defecation function between TAMIP‐IPAA and traditional IPAA. The TAMIP‐IPAA group demonstrated superior outcomes with lower Wexner scores and improved defecation frequency, shedding light on the functional advantages of the TAMIP approach.

The favorable postoperative defecation function outcomes with TAMIP are due to its precise mucosa excision, preserving both internal and external anal sphincter muscles. In addition, more accurate pelvic dissection avoids trauma and allows safe distal rectal dissection, especially in men with a narrow pelvis.^4^ In contrast, traditional IPAA, with mucosal dissection performed under direct vision, especially in areas distant from the anus, poses risks for sphincter muscle damage.

Maintaining an optimal rectal cuff length is crucial for pouch function. The TAMIP approach allows for precise adjustment of the rectal cuff length, unlike stapled IPAA, which may encounter difficulties in adjusting the remaining rectum length, or traditional IPAA, which have difficulties in mucosal‐only excision under direct visualization in areas distant from the anus.

Economically, stapled IPAA and TAMIP‐IPAA have similar surgical expenses, slightly higher than traditional IPAA. However, due to higher complication rates and longer postoperative hospital stays, the median hospitalization costs for traditional IPAA were 1.3 times higher than for TAMIP‐IPAA. Therefore, the TAMIP approach not only reduces complications but also lowers the overall financial burden on patients.

Despite its advantages, TAMIP requires careful consideration due to potential complications, such as urethral injury.^9^ In this study, there were no cases of urethral injury or urinary dysfunction. In general, the transanal approach requires systematic training and a long learning curve because it differs from the normal anatomical field of view.^9^ In our department, to ensure technical quality, surgeons must first gain experience as camera assistants during five or more cases before becoming transanal approach surgeons, and we take steps to familiarize them with the transanal field of view.

The rates of postoperative complications, readmissions, and pouch failures were notably higher in the non‐TAMIP, especially in the traditional IPAA, compared to the TAMIP group. However, due to differences of the background and surgical procedure, our data are compared with previous reports in Table 5.

**TABLE 5:** Comparison of patient background, operative complications, and functional outcome with previous research.

Author	Disease	*n*	Age*	Use of TNFα	Use of steroid	Approach/construction method	Operative time* (min)	Blood loss* (mL)	CD Grade	AL	SBO	Post‐op Hospital stay* (day)	Readmission	Follow‐up* (years)	Pouch failure
Saigusa N, et al^31^ 2000	UC/FAP	3	32.7	N.A.	N.A.	Open/IPAA (Handsewn/stapled)	N.A.	N.A.	N.A.	N.A.	N.A.	N.A.	N.A.	N.A.	N.A.
Kawamura J, et al^23^ 2013	UC	28	35	0	46.0%	Lap/TA‐IPAA (Handsewn)	440	80–140	≥II 57.1% ≥III 17.9%	3.6%	42.9%	N.A.	N.A.	N.A.	7.1%
Tasende MM, et al^19^ 2015	UC	16	N.A.	N.A.	N.A.	Lap/IPAA (Handsewn/stapled)	170	N.A.	Total 37.5%	0.0%	6.3%	7	N.A.	1.9	0%
C. A. Leo, et al^4^ 2015	UC	16	46	0	12.5%	Lap/IPAA (Handsewn/stapled)	247	N.A.	Total 43.8%	6.2%	25.0%	6	N.A.	N.A.	N.A.
P. Ambe, et al^21^ 2017	FAP	8	19	0	0.0%	Lap/TAn IPAA (stapled)	470	N.A.	Total 12.5%	25.0%	N.A.	13	12.5%	N.A.	12.5%
de Buck van Overstraeten, et al^7^ 2017	UC/IBDU	216	37	13.4%	9.7%	Lap or Robot/IPAA TAn 97 TA 119	218	N.A.	≥II 1.9% ≥III 17.5% (TAn)	7.4%	N.A.	TA 9, TAn 7	N.A.	N.A.	N.A.
Lask A, et al^20^ 2021	UC	22	32	N.A.	N.A.	Lap/Tan‐IPAA (Handsewn/stapled)	N.A.	N.A.	≥II 22%	9.0%	N.A.	N.A.	N.A.	1.3	21.4%
Park L, et al.^8^ 2022	UC	113	36.5	11.2%	N.A.	Lap/Tan‐IPAA (Handsewn/stapled)	320	N.A.	≥II 43.4% ≥III 18.4%	Handsewn 14.3%, Stapled 5%	21.1%	TA4, TAn 3.3	TA 27%, TAn 26%	N.A.	0.9%
Fukui R, et al^32^ 2023	UC/FAP	32	50.5	N.A.	N.A.	Lap/IPAA (Handsewn/stapled)	N.A.	N.A.	≥II 77.8% ≥III 16.7%	N.A.	N.A.	N.A.	N.A.	4.6	31.3%
Current study	UC	37	35	67.6%	73.0%	Lap/TAn‐IPAA (TAMIP)	289	130	≥II 29.7% ≥III 8.1%	8.1% (Handsewn)	10.8%	8	8.1%	4.0	8.2%
Current study	UC	7	52	71.4%	71.4%	Lap/TA‐IPAA	443	392	≥II 57.1% ≥III 28.6%	42.9% (Handsewn)	28.6%	18	57.1%	7.0	28.6%

Abbreviations: AL, anastomotic leakage; CD, Clavien–Dindo; FAP, familial adenomatous polyposis; IBDU, inflammatory bowel disease unclassified; IPAA, ileal pouch‐anal anastomosis; Lap, laparoscopic surgery; Ro, robot‐assisted surgery; SBO, small bowel obstruction; TA, transabdominal; TAMIP, transanal minimally invasive proctectomy; TAn, transanal; UC, ulcerative colitis.

* Median.

Anastomotic leakage rates have been reported between 0% to 25%,^4^, ^7^, ^8^, ^19^, ^20^, ^21^, ^22^, ^23^ indicating that the incidence in TAMIP‐IPAA falls within or slightly higher, while traditional IPAA shows a notably higher incidence (Table 5). A disease duration of UC over 5 years and concurrent use of anti‐TNFα and steroids are known independent risk factors for anastomotic leakage.^22^ In our study, the median UC duration was 6 years in the TAMIP‐IPAA group and 18 years in the traditional IPAA group. Additionally, 65% of the TAMIP‐IPAA and 71.4% of the traditional IPAA group used both medications. Our institution frequently treats patients with long UC durations and multiple treatments, suggesting both cohorts may have a higher risk for anastomotic leakage than previously reported.

TAMIP group showed comparable outcomes regarding bowel obstruction and re‐admission rates, while the traditional IPAA group had a higher incidence than previously reported (Table 5). In our study, the majority of small bowel obstruction cases were due to stoma outlet obstruction (SOO), which reported particularly common after laparoscopic surgery.^23^ Given that all cases in this study were laparoscopic, the higher frequency of SOO warrants careful interpretation and highlights the need for future studies to address this issue.

The reasons of re‐admission in the non‐TAMIP group included small bowel obstruction (two cases), delayed anastomotic leakage (two cases), and bleeding from a duodenal ulcer (one case), totaling five cases (14.7%). In the TAMIP group, all three readmissions (8.1%) were due to dehydration from high output. The shorter postoperative hospital stay in the TAMIP group, facilitated by Enhanced Recovery After Surgery (ERAS) protocols, may have contributed to fluid management challenges upon discharge. Such patients require more stringent follow‐up in the outpatient setting, which represents a key area for future improvement. Two patients in the non‐TAMIP group were readmitted due to delayed anastomotic leakage. As previously noted, this is likely attributable to the higher predisposition to anastomotic leakage within this cohort.

Regarding pouch failure, the rate in the TAMIP group (three out of 37, 8.2%) was consistent or slightly higher, while the traditional IPAA group had obviously higher rate (two out of seven, 28.6%) than previous reports (Table 5). In the traditional IPAA group, the two cases of pouch failure occurred in patients aged 52 and 62, aligning with data suggesting an increased risk in older patients.^24^, ^25^ In the TAMIP group, two out of the three pouch failure cases have risk factors such as perioperative steroid use (≥40 mg/day) and severe pelvic sepsis arising from severe pouchitis,^26^ which makes redo surgery challenging and results in the creation of a stoma.

Our findings suggest that TAMIP‐IPAA may offer enhanced postoperative outcomes and defecation function compared to traditional IPAA, particularly crucial for the younger population of patients with UC.^27^ Whereas compared to previous reports, our TAMIP approach demonstrated comparable outcomes overall, with a slightly higher incidence of anastomotic leakage and pouch failure. This may be attributed to the higher proportion of UC patients with extensive treatment histories, however, accumulating more cases will be essential for further evaluating the efficacy of TAMIP.

This report has some limitations. Firstly, being a single‐center retrospective cohort study may introduce biases. Additionally, an uneven case distribution, with seven cases for traditional IPAA and 37 for TAMIP‐IPAA, could impact generalizability. Notably, traditional IPAA cases were from an earlier era, and no assessment based on different eras was conducted. Advancements in laparoscopic technology and increased surgeon proficiency may have contributed to the shorter operative time in the TAMIP approach. The implementation of ERAS protocols and clinical pass revisions, emphasizing early discharge, likely resulted in a shorter postoperative hospital stay, particularly in the TAMIP group, which has more readily adopted these innovations.

The absence of preoperative defecation function assessment hinders pre‐ and post‐surgery outcome comparisons. Administering preoperative questionnaires, especially in emergency UC cases, may face time constraints. Even in non‐emergency cases, severe symptoms or resistance to medical treatment can affect questionnaire reliability. Additionally, recall bias is likely in patients asked about their defecation function before the implementation of a standardized questionnaire system. This represents a major limitation of the study.

In the TAMIP approach, the inherently enhanced efficiency, facilitated by two teams, contributes to smoother procedure progression. However, the number of surgical staff may also influence operative time.^28^

The analysis of the learning curve for traditional IPAA suggests that over 200 cases may be required,^29^ while TAMIP‐IPAA needs at least 20–40 cases.^30^ Given these differences, the TAMIP approach may have facilitated a relatively rapid reduction in operative time.

There was a significant difference in age at surgery between the non‐TAMIP and TAMIP groups, likely influencing postoperative complications^25^ and pouch failure.^24^ In addition, the surgical procedure varies with age, as a three‐stage surgery is often adopted for elderly patients due to its invasiveness. Future studies should address these limitations for a comprehensive evaluation of TAMIP efficacy.

## 5 CONCLUSION

The TAMIP approach during proctocolectomy for UC has the potential to offer promising benefits, including the enhancement of short‐term outcomes and the improvement of defecation function. This suggests a compelling advantage in the comprehensive management of UC.

## AUTHOR CONTRIBUTIONS

M.H. designed the study, the main conceptual ideas, and the proof outline. Y.K. made critical revision. K.Y., A.T, M.S., S.Y. collected the data. M.T. and Y.K. made a final approval of the manuscript. All authors read and approved the final manuscript.

## FUNDING INFORMATION

No funding was received for this study.

## CONFLICT OF INTEREST STATEMENT

Author Y.K. is an Associate Editor of *Annals of Gastroenterological Surgery*, and received speaker honoraria from Intuitive Surgical, Johnson & Johnson KK, and Medtronic Japan. Author M.T. received speaker honoraria from Johnson & Johnson KK, Medtronic Japan, Olympus, and Intuitive Surgical. The other authors have no conflicts of interest, funding, or other sources of support to declare in connection with the submitted article. The funding source had no role in the design, practice, or analysis of this study.

## ETHICS STATEMENTS

The protocol for this research project has been approved by a suitably constituted ethics committee of the institution and it conforms to the provisions of the Declaration of Helsinki (Ethics Committee of Tokyo Medical and Dental University, M2020‐367). All informed consent was obtained from the subjects.
